# Editorial: What's Shared in Sharing Tasks and Actions? Processes and Representations Underlying Joint Performance

**DOI:** 10.3389/fpsyg.2019.00659

**Published:** 2019-04-02

**Authors:** Motonori Yamaguchi, Timothy N. Welsh, Karl Christoph Klauer, Kerstin Dittrich

**Affiliations:** ^1^Department of Psychology, Edge Hill University, Ormskirk, United Kingdom; ^2^Faculty of Kinesiology & Physical Education, University of Toronto, Toronto, ON, Canada; ^3^Department of Psychology, Albert-Ludwigs-Universität Freiburg, Freiburg, Germany

**Keywords:** joint task, joint action, task sharing, co-representation, joint cognition

## Six Blind Men and an Elephant

There is an ancient story about six blind men who met to discuss what an elephant is like. Each of them touched a different part of the elephant and came up with an image of what an elephant was like. The first man touched its leg and thought that an elephant was like a pillar; the second man touched its ear and thought that an elephant was like a fan; the third man touched its trunk and thought that an elephant was like a snake, and so on. The six blind men, the six different images of what an elephant was like. The six men quarreled over an elephant, until they realized that they touched different parts of the same elephant. How can these blind men ever learn what an elephant is really like? Doing so requires integrating other people's images of an elephant into their own, the process known as *co-representation*.

The story of six blind men and an elephant offers several morals. One of the morals is that it is very difficult to see the whole from its parts, especially when the parts are distributed among different individuals. This poses a challenge that individual actors face when they share a task with co-actors, and it is important to understand cognitive mechanisms that meet the challenge. The present research topic aimed at bringing together different approaches and perspectives to the study of sharing tasks and actions between co-acting individuals. It was hoped that these perspectives would collectively present a big picture that delineates the cognitive processes and representations underlying joint performance.

## The Beginning: Sharing Tasks and Actions

The last decade has seen a surge of interest in experimental studies of joint task performance. These studies have suggested that actors who share a single task not only perform it together, but also share a mental representation of the whole task; that is, actors co-represent both their and their co-actors portion of the task. Initial evidence supporting the idea of co-representation was garnered through the *joint Simon task* (Sebanz et al., 2003), wherein pairs of actors divide the work involved in performing a choice reaction task. In the standard version of the Simon task with a single actor, response times (RT) are shorter when the responses spatially correspond to the location of stimuli (e.g., pressing a left key to circles on the left side of a computer monitor) than when they do not (pressing a left key to circles on the right). This Simon effect disappears if the task setting is altered in such a way that spatial attributes of stimuli or choice of responses are eliminated. For example, in a go/nogo version of the task, the actor responds to a type of stimulus (e.g., red circles) by pressing one key and withholds responding to another type of stimulus (green circles). The Simon effect disappears in this task context because only a single response is involved in the task, so the spatial attribute is no longer used to represent the response and there is no response conflict to resolve. However, the Simon effect re-emerges when two actors perform the go/nogo version of the task together. In this joint Simon task, each co-actor operates one of the two response keys to respond to one type of stimulus, and is told to withhold a response when the stimulus assigned to their partner is presented. The critical finding of these studies is that RTs are still shorter if stimuli occur on the same side as the response location than if they occur on the opposite side. This finding has been used to argue that co-acting individuals co-represent (or share a mental representation of) the task.

## Thirteen Articles, Thirty-Seven Authors, and One Conclusion?

More than a decade after the original study, the joint Simon task still remains to be a popular paradigm of task sharing, but in more complex situations that involve multiple modalities (Dolk and Liepelt) or a large number of display items (Baess et al.) and with more elaborate measures, such as autocorrelation (Ciardo and Wykowska) and sequential modulations (Mendl et al.) of RTs as well as event-related potentials (Michel et al.). Other behavioral paradigms have also been developed to study interpersonal phenomena in a joint task setting, such as attentional blink in the rapid sequential visual presentation (Constable et al.), four alternative forced choice (Czeszumski et al.), line bisection (Dosso et al.), stimulus and response priming in a prime-probe task (Giesen et al.), and Stroop interference (Yamaguchi et al.). As in the Simon task, these paradigms measure discrete actions (e.g., pressing a key), but paradigms that require continuous actions have also made important contributions to our understanding of joint performance (Ray and Welsh; Rocca and Cavallo; Wahn et al.).

Studies of task sharing now demonstrate a variety of issues in joint tasks and actions. Several groups investigated the influences of interpersonal relationships on joint tasks and actions (Ciardo and Wykowska; Czeszumski et al.; Giesen et al.; Mendl et al.) while others examined the influences of joint settings on the frame of reference (Baess et al.; Dolk and Liepelt; Dosso et al.; Ray and Welsh). Although most studies in this collection focused on co-representation (integration) between co-acting individuals, others pointed out the importance of a division of labor in task sharing (Constable et al.; Wahn et al.; Yamaguchi et al.). The neural basis of joint task performance is still an under-investigated area of study (Czeszumski et al.; Michel et al.) that requires further development in future research.

The present collection includes 13 articles by 37 authors. What these studies tell us about task sharing? In a nutshell, most studies in the present collection found little evidence for co-representation (Baess et al.; Constable et al.; Dolk and Liepelt; Dosso et al.; Michel et al.; Yamaguchi et al.) or limited support for co-representation that was conditional on the interpersonal relationship with the co-acting partners (Ciardo and Wykowska; Giesen et al.; Czeszumski et al.; Mendl et al.; Ray and Welsh).

After the initial demonstration of co-representation (Sebanz et al., 2003), a large number of studies have explored conditions under which co-representation occurs or does not occur (e.g., Welsh et al., 2009; Dittrich et al., 2013; Yamaguchi et al., 2018). These efforts have enriched the empirical ground to understand cognitive processes and representations underlying joint task performance, and alternative accounts of task sharing have been proposed (e.g., Dittrich et al., 2012; Dolk et al., 2014; Prinz, 2015; Yamaguchi et al., 2019). The present collection adds further empirical evidence to aid such efforts. They imply that the six blind men still have difficulty seeing what a real elephant is like, but we have started to understand why it is so difficult to see the elephant.

## Author Contributions

MY wrote the first draft, and all authors contributed to revisions and approved the final version for publication.

### Conflict of Interest Statement

The authors declare that the research was conducted in the absence of any commercial or financial relationships that could be construed as a potential conflict of interest.
